# Dynamical Synergy of Drug Combinations during Cancer Chemotherapy

**DOI:** 10.3390/jpm12111873

**Published:** 2022-11-09

**Authors:** Paolo Castorina, Emanuele Martorana, Stefano Forte

**Affiliations:** 1 INFN, Sezione di Catania, I-95123 Catania, Italy; 2 Faculty of Mathematics and Physics, Charles University, V Holešovičkách 2, 18000 Prague, Czech Republic; 3 Istituto Oncologico del Mediterraneo, 95029 Viagrande, Italy

**Keywords:** synergy, drug combination, chemotherapy, tumor progression

## Abstract

Synergistic drug combinations often provide effective strategies to increase treatment efficacy and, during therapy, it is a time-dependent process. Data for colorectal and lung cancer in vivo were used for the phenomenological study of dynamical synergy during treatments. The proposed approach takes into consideration tumor regrowth by macroscopic laws. The time dependencies of synergistic drug combinations are analyzed by different parametric indicators. The cumulative effects of the single therapy and drug combinations are quantitatively well described and related to the cumulative doses. In conclusion, the analysis of dynamical synergy during chemotherapy has to take into account the effects of the drug doses and the tumor regrowth, which can provide a reliable description of the synergistic time dependence.

## 1. Introduction

Synergistic drug combinations often provide effective strategies to increase treatment efficacy. Computation analyses of drug synergism are therefore of relevance for quantitatively understanding the effects of known and new drug combinations and drug-target interactions.

Drug synergism has been studied for many different diseases (such as infectious fungi [1,2,3], osteoarthritis [4], hypertension [5,6], cancer growth (see for example [7]), and large parts of the experimental/phenomenological/theoretical research studies that focus on the dose–effect curve and determine the synergistic results according to the specific definition of synergy and computational methods [8,9,10,11,12,13]. Drug-target interactions, drug chemical similarity, and data analyses from biological experiments were studied by network-based models, such as the protein–protein interaction networks and machine learning approaches [13].

All previous important studies on synergy concern the relation between fixed, specific drug doses and the corresponding effects. On the other hand, in general, drug therapy is a dynamic process with dose administrations at regular intervals (daily, weekly, etc.) for a period of time. For example, cancer chemotherapy protocols usually involve drug administrations for about 3 weeks. Accordingly, the synergistic effect could be time-dependent during cancer therapy; this aspect is phenomenologically discussed in this paper, by an approach that takes into account the regrowth pattern.

The analysis performed in the following sections is based on in vivo data for colon–rectal and lung cancer reported in [14] and on macroscopic tumor growth laws. The synergy of drug combinations is time-dependent; moreover, we discuss the correlation with the cumulative doses during therapy.

## 2. Material and Methods

### 2.1. Time-Dependent Drug Synergism during Chemotherapy: Data Analysis

Dynamical drug synergism during chemotherapy has been verified by in vivo data taken from the MMHCdb repository [14]. We focus on colorectal and lung cancer, as they are the most common non-sex-specific deadly cancers [15]; we extracted data from the patient-derived xenograft model (PDX). The data concern the primary tumor; therefore, the metastatic PDX was not considered. The experiments showed tumor volume evolution within 28 days using a protocol for the dosage and measurement of the drug response. In the analyzed data, six different drugs were administrated. The platinum-based chemotherapy drugs, cisplatin, and oxaliplatin, are typically administered to treat various types of tumors, in particular for colorectal cancer. Compared to cisplatin, with oxaliplatin, the two amine groups are replaced by cyclohexyl diamine for improved antitumor activity. Docetaxel is a clinically well-established anti-mitotic chemotherapy medication used mainly for the treatment of breast, ovarian, and non-small cell lung cancer. Trametinib dimethyl sulfoxide is a kinase inhibitor. Understanding the mechanism of action of 5-fluorouracil (5-FU) has led to the development of strategies that improve its anticancer activity. Despite these advances, drug resistance remains a significant limitation to the clinical use of 5-FU. Table 1 summarizes some characteristics of the PDX and the applied dosing study.

The dosing study for the lung and colon cancer model involved 40 and 45 mice, respectively, divided equally into 5 groups. The treatment and time point of each group are described in Table 2. The details of the dosing protocols per group are given below.

Lung cancer dosing protocol:
  - CMC plus D5W (placebo treatment no. 1) (0.5% carboxymethyl cellulose +5% dextrose water) 5 mL/kg intravenous weekly (three times), and 10 mL/kg by oral gavage daily for 21 days;
  - Cisplatin (treatment no. 2) 2 mg/kg intravenous weekly (three times);
  - Docetaxel (treatment no. 3) 6 mg/kg intravenous weekly (three times);
  - Trametinib (treatment no. 4) 2 mg/kg oral garage daily for 21 days;
  - Trametinib plus docetaxel (treatment no. 5) 2 mg/kg by oral gavage daily for 21 days and 6 mg/kg intravenous weekly (three times)
Colon cancer dosing protocol
  - D5W ((placebo treatment no. 1) (5% dextrose water) 5 mL/kg intravenous weekly (three times);
  - 5-FU (treatment no. 2) 20 mg/kg intravenous weekly (three times);
  - Cisplatin (treatment no. 3) 2 mg/kg intravenous weekly (three times);
  - Oxaliplatin (treatment no. 4) 5 mg/kg intraperitoneal weekly (three times);
  - Oxaliplatin plus 5-FU (treatment no. 5) 5 mg/kg intraperitoneal and 20 mg/kg intravenous weekly (three times).

**Figure 1:**
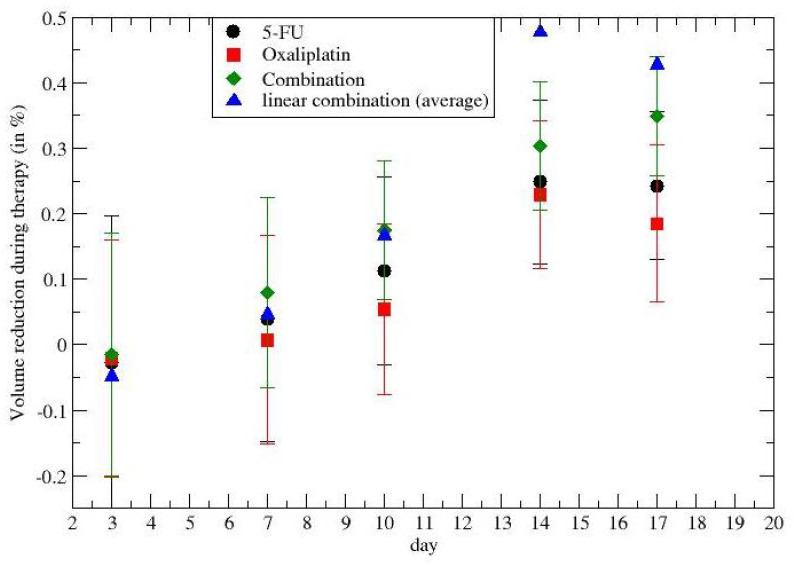
Time dependence of the drug synergism in colon–rectal cancer chemotherapy. Day 1 is the day before the first drug administration at t=0.

The reduction of the gross tumor volume during chemotherapy for colon–rectal cancer (for different drug administrations) is depicted in Figure 1. The reported quantity is the ratio

(1) $$R=\frac{V_{nd}-V_{d}}{V_{nd}}$$

as a function of time, where $V_{nd},V_{d}$ are, respectively, the gross tumor volumes without and with therapy. Black points describe the effect of the 5-FU drug, the red points concern oxaliplatin, and the green points are the synergistic results. The blue triangles were obtained by a linear superposition of the two (independent) drug effects.

**Figure 2:**
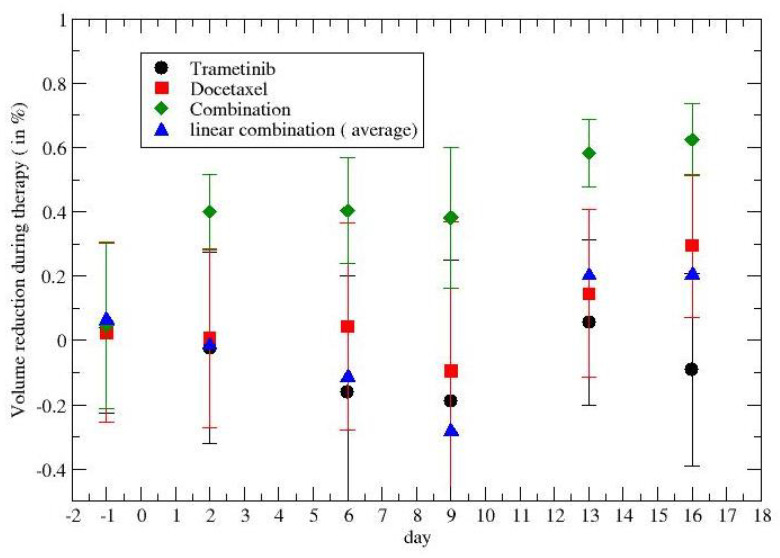
Time dependence of the drug synergism in lung cancer chemotherapy. Day 1 is the day before the first drug administration at t=0.

The analogous results for lung cancer are given in Figure 2. Black points describe the effects of trametinib, the red points concern docetaxel, and the green points are the synergistic results. The blue points were obtained by a linear superposition of the two (independent) drug effects.

The time dependence of the synergy is clear and its quantitative description will be discussed in the following section, taking into account the tumor regrowth.

### 2.2. Time-Dependent Drug Synergism during Chemotherapy: Quantitative Description

The Gompertz law (GL, see Appendix A) [16], initially applied to human mortality tables (i.e., aging), describes tumor evolution ( for a recent review, see [17]). It depends on two parameters, which for cancer are related to the initial exponential trend and to the maximum number of cells, $N_{\infty }$, called the carrying capacity, and can be supported by the local micro-environmental conditions (angiogenesis, immune system activity, nutrient supply, hypoxia, etc.). For homogeneous systems, $N_{\infty }$ corresponds to the volume carrying capacity, $V_{\infty }$; we consider such systems, although the approach can be easily generalized to non-homogeneous ones. For untreated tumors, the GL for the volume V(t) is the solution of the differential equation (see Appendix A)

(2) $$\frac{1}{V}\frac{dV}{dt}=kln\left(\frac{V_{\infty }}{V}\right).$$

In the cell-killing model of chemotherapy (see, for example, [18]), a new term, representing the therapy effect, is included in the second term of Equation (1), i.e.,

(3) $$\frac{1}{V}\frac{dV}{dt}=kln\left(\frac{V_{\infty }}{V}\right)-c\left(t\right)$$

where c(t) is the rate of the specific volume reduction due to the drug treatment. The general solution is

(4) $$V_{d}\left(t,t_{0}\right)=V_{nd}\left(t,t_{0}\right)-I\left(t,t_{0}\right)$$

where

(5) $$I\left(t,t_{0}\right)=\int_{t_{0}}^{t}dt^{{}^{\prime }}c\left(t^{{}^{\prime }}\right)e^{-k(t-t^{{}^{\prime }})}.$$

For $t_{0}=0$, one obtains the cumulative effect of the therapy, I(t,0).

## 3. Results

**Figure 3:**
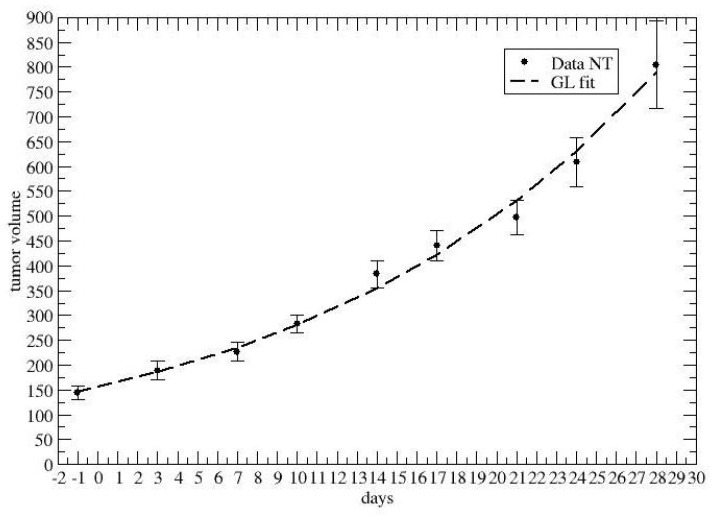
GL fit of the colon–rectal tumor growth without chemotherapy.

**Figure 4:**
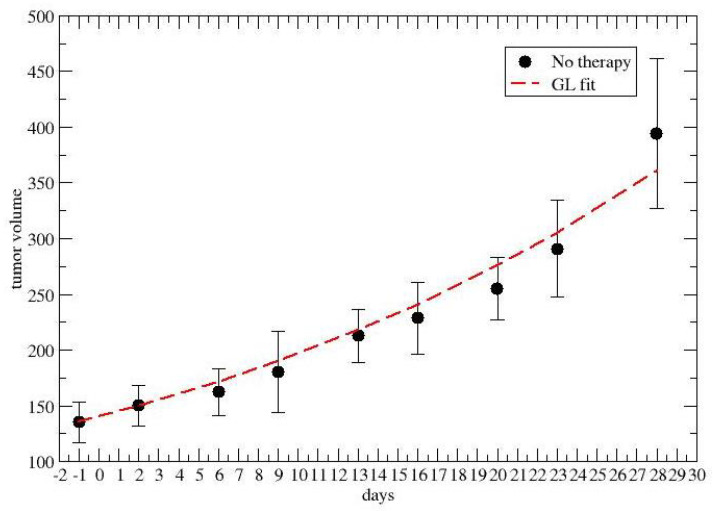
GL fit of the lung tumor growth without chemotherapy.

Firstly, Figure 3 and Figure 4 report the GL fits of the control groups, i.e., no chemotherapy, for colon–rectal and lung cancer, respectively.

**Figure 5:**
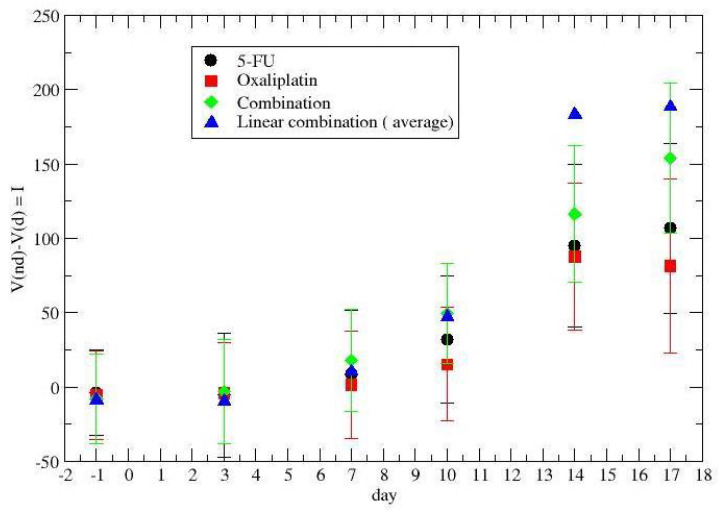
The cumulative effects of chemotherapy for different drugs and their combinations for colon rectal cancer. Day 1 is the day before the first drug administration at t=0.

**Figure 6:**
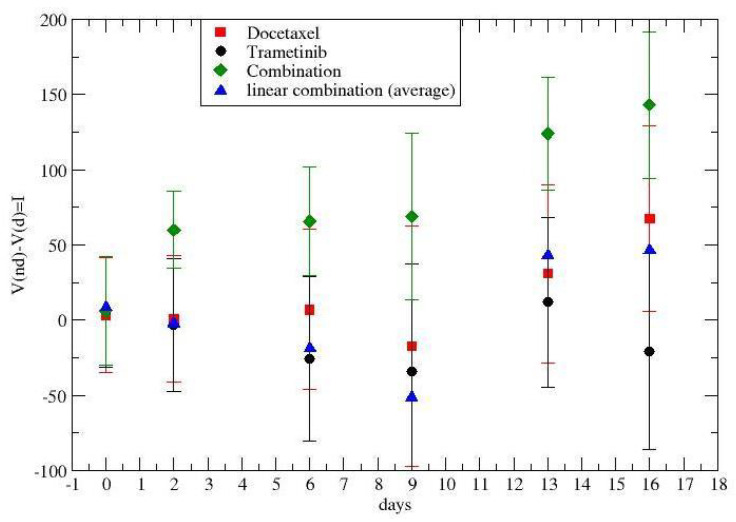
Cumulative effects of chemotherapy for different drugs and their combinations for lung cancer. Day 1 is the day before the first drug administration at t=0.

The cumulative effect of the therapy, i.e., I(t,0), is in Figure 5 for colon–rectal cancer and in Figure 6 for lung cancer. The time-dependence synergy is due to the combination of tumor regrowth and therapy.

From the average values, I(t,0), one can derive the average rate c(t). Indeed, for colon–rectal cancer, the synergistic value is easily fitted by $I\left(t,0\right)=\alpha t^{2}$ with α=0.544 in day${}^{-2}$ and, therefore, by Equation (5), one has

(6) $$c\left(t\right)=\beta t+\delta t^{2}$$

with β=2δ/k and α=δ/k. Therefore, during therapy, the cell-killing fraction grows according to Equation (6) up to the critical value $t^{*}$, such that the sign of the specific growth rate in the GL (second term in Equation (3)) becomes negative, i.e.,

(7) $$c_{crit}=c\left(t^{*}\right)=kln\left(\frac{V_{\infty }}{V(t^{*})}\right)$$

Analogously, for the single drugs, the behavior is $I_{5-FU}=\alpha_{\left[5-FU\right]}t^{2}=0.4t^{2}$ and $I_{Oxa}=\alpha_{Oxa}t^{2}=0.32t^{2}$.

Previous Figure 1, Figure 2, Figure 5, and Figure 6 show that the critical value, i.e., the fixed point of Equation (3), is not reached, since the average derivative, dV/dt, is always positive (the few negative values are due to the experimental errors, since one observes an increasing trend).

The combined effects can also be evaluated by considering the average specific growth rate in the interval between two subsequent drug doses, i.e.,

(8) $$\frac{1}{\Delta t}\frac{V(t+\Delta t)-V(t)}{V(t)}$$

where Δt is the interval and t=0,Δt,2Δt,3Δt, etc.

For colon–rectal cancer, for example, the results are reported in Table 3, where the reduction of the specific growth rate (with respect to the non-therapy case) clearly shows the effects of the single drugs and their combinations. Of course, the rate is positive since the critical values were not reached.

## 4. Discussion and Conclusions

Synergy during chemotherapy is a pharmacodynamic process. Indeed, the time interval of the therapeutic effect of a single dose is unclear; for example, the first dose gives almost no contribution to the volume reduction at the end of the treatment. Moreover, there is no way to determine a priori the maximum effect and the potency (i.e., the dose that produces 50% of the maximum). On the other hand, a possible indication can be obtained by considering the relation between the cumulative effect I(t,0) and the cumulative dose D(t) at time *t*. To quantify this point, let us consider the colon–rectal cancer data. The single doses of 5−FU, $d_{1}$, Oxaliplatin, $d_{2}$, and the combination, $d=d_{1}+d_{2}$, are given at time t=0,7,14 (in the day). The corresponding cumulative doses are $D_{1}\left(t\right)=d_{1}*\left(t/\Delta +1\right),D_{2}\left(t\right)=d_{2}*\left(t/\Delta +1\right),D\left(t\right)=d*\left(t/\Delta +1\right)$, with Δ=7 in the day. By assuming, for the sake of simplicity, a continuous relation between *t* and the cumulative dose, the effect is related to the square of the dose, i.e.,

(9) $$I\left(t,0\right)≃D{\left(t\right)}^{2}$$

which recalls the Hill equation with the slope parameter p=2 and in the region of the low dose, i.e., $D^{2}<<$ potency (see, for example, [10]). More precisely,

(10) $$I\left(t,0\right)=\Delta^{2}\alpha {\left(\frac{D}{d_{1}+d_{2}}-1\right)}^{2}$$

(11) $$I{\left(t,0\right)}_{5-FU}=\Delta^{2}\alpha_{\left[5-FU\right]}{\left(\frac{D_{1}}{d_{1}}-1\right)}^{2}$$

(12) $$I{\left(t,0\right)}_{Oxa}=\Delta^{2}\alpha_{Oxa}{\left(\frac{D_{2}}{d_{2}}-1\right)}^{2}$$

In conclusion, the analysis of synergy during chemotherapy has to take into account (at least) two aspects: the effect of the drug doses and the tumor regrowth. To quantify these dynamical elements, we considered the GL and the modification of the specific rate due to the treatment (c(t) in the second term of Equation (3)).

We have shown that c(t) is an increasing function of time; however, it does not reach the critical value of the specific rate change sign, producing a depletion of the tumor size. Pharmacodynamic synergy, including regrowth, can be described by the function I(t,0) for the different treatments, by the time dependence of the corresponding functions c(t), and by the discretization of the specific rate. The proposed approach is general and helpful for understanding new drug combinations and drug–target interactions, although it should be confirmed by a larger in vivo data sample and considering other tumor phenotypes before its application to human trials.

## Figures and Tables

**Table 1:** Summary description of the PDX models used in our work. From left to right we specify the tumor body location, the origin of the tumor (primary or metastatic), histologic classification, the score assigned using the American Joint Committee on Cancer (AJCC) staging system, and the name of the chemo drug administered.

PDX Characteristics				
**Tumor Site**	**Type**	**Diagnosis**	**Stage (AJCC)**	**Chemotherapy**
Lung	Primary	Mucinous adenocarcinoma	IV	CMC plus D5W Cisplatin Docetaxel Trametinib Docetaxel plus Trametinib
Colorectal	Primary	Adenocarcinoma	IV	D5W Cisplatin 5-FU Oxaliplatin Oxaliplatin plus 5-FU

**Table 2:** Schedule of the treatments and measurements for the lung and colon PDX model. The symbol X*, when specified for combined treatments, represents the administration of both doses, otherwise, the given drug is the first listed in the dosing protocols.

Treatments and Measurements Schedule												
**Days**	**PDX Lung**						**PDX Colon**					
	**Treatments**					**Meas.**	**Treatments**					**Meas.**
	#1	#2	#3	#4	#5		#1	#2	#3	#4	#5	
−1						X						X
0	X*	X	X	X	X*		X	X	X	X	X	
1	X			X	X							
2	X			X	X	X						
3	X			X	X							X
4	X			X	X							
5	X			X	X							
6	X			X	X	X						
7	X*	X	X	X	X*		X	X	X	X	X	X
8	X			X	X							
9	X			X	X	X						
10	X			X	X							X
11	X			X	X							
12	X			X	X							
13	X			X	X	X						
14	X*	X	X	X	X*		X	X	X	X	X	X
15	X			X	X							
16	X			X	X	X						
17	X			X	X							X
18	X			X	X							
19	X			X	X							
20	X			X	X	X						
21												X
22												
23												
24												X
25												
26												
27												
28												X

**Table 3:** The specific growth rate per day in the time interval between doses, including the regrowth rate and colon–rectal cancer.

TimeInterval-Days	No Therapy	5-FU	Oxap.	Combination
0–7	0.081	0.067	0.071	0.053
7–14	0.098	0.047	0.034	0.04
14–21	0.043	0.051	0.053	0.037

## Data Availability

All data are available upon request, please contact the corresponding author.

## Abbreviations

The following abbreviations are used in this manuscript:

GL	Gompertz law
PDX	patient-derived xenograft
CC	carrying capacity
GTV	gross tumor volume
5-FU	5-fluorouracil
CMC	carboxymethyl cellulose
D5W	5% dextrose water

## Appendix A

The GL, for homogeneous systems, is a solution to the equation

(A1) $$\frac{1}{V}\frac{dV}{dt}=kln\left(\frac{V_{\infty }}{V}\right),$$

where $V_{\infty }$ is the volume CC. Therefore, the GTV evolves in time according to

(A2) $$\frac{V(t)}{V(t_{0})}=e^{ln\left(\frac{V_{\infty }}{V_{0}}\right)\left[1-exp\left(-k\left(t-t_{0}\right)\right)\right]}$$

where $V_{0}$ is the initial ($t_{0}$) value. It is useful to clarify that, in some cases, one needs a long time series of observations to disentangle the GL behavior by an exponential one. Indeed, if $k(t-t_{0})<<1$, the solution can be approximated as

(A3) $$\frac{V(t)}{V(t_{0})}=e^{ln\left(\frac{V_{\infty }}{V_{0}}\right)*k\left(t-t_{0}\right)}$$

which is an exponential decrease (recall that $V_{\infty }/V_{0}<1)$. In such cases, the independent determinations of the two GL parameters, $V_{\infty },k$ cannot be conducted unambiguously since the $\chi^{2}$ is extremely flat in a large parametric region, and only the product $ln(\frac{V_{\infty }}{V_{0}})*k$ can be obtained by fitting the experimental data.
